# Morphology

**Published:** 1885-12

**Authors:** 


### Morphology.

Mr. Paul Albrecht makes some startling identifications of homologies between parts of the mammalian skull and elements present in the lower vertebrates.

By a course of reasoning based upon the examination of skulls, in which ossification was defective, this anatomist has found the quadrate bone of reptiles to be present in mammals in its normal position but to synostose with the squamosal early in life. Another result of this course of reasoning is the homologizing of the teleostean parasphenoid with that part of the basi-post sphenoid which is not included in the clivus, and another, that the trigeminal nerve of the mammalia leaves the true cranium at the same spot which it pierces in the non-mammalian gnathostomes. The mere fact that the symplectic gives origin to the cartilage of Meckel shows that it must belong to the mandibular arch.

M. Albrecht maintains that the metaphrygoid (squamosal) quadrate ectophrygoid (alisphenoid) entophrygoid and palatines form a premandibular visceral arch or rib. From this it follows, that the spiracles (of selachians) and the eustachian tube are morphologically a premandibular bronchial sac.—*American Naturalist.*

Menthol as a Local Anæsthetic.—Rosenberg (*Berliner klin. Wochenschr.*; Lancet) finds that a twenty or thirty per cent. solution of menthol, which is much cheaper than cocaine, is a useful substitue for the latter as an anæsthetic application to mucous surfaces, like those of the nose, the pharynx, and larynx. Although its effect is more evanescent than that of cocaine, it appears somewhat cumulative, for, when repeated, even after a long interval, the latter application produces a longer period of anæsthesia than the earlier one.—*New York Medical Journal.*

